# A 3D Finite Element Analysis Model of Single Implant-Supported Prosthesis under Dynamic Impact Loading for Evaluation of Stress in the Crown, Abutment and Cortical Bone Using Different Rehabilitation Materials

**DOI:** 10.3390/ma14133519

**Published:** 2021-06-24

**Authors:** Oriol Cantó-Navés, Raul Medina-Galvez, Xavier Marimon, Miquel Ferrer, Óscar Figueras-Álvarez, Josep Cabratosa-Termes

**Affiliations:** 1 Faculty of Dentistry, Universitat Internacional de Catalunya (UIC), 08017 Barcelona, Spain; oriolcanto@uic.es (O.C.-N.); ruldoc@uic.es (R.M.-G.); ofigueras@uic.es (Ó.F.-Á.); cabratosa@uic.es (J.C.-T.); 2 Bioengineering Institute of Technology, Universitat Internacional de Catalunya (UIC), 08190 Barcelona, Spain; 3 Automatic Control Department, Universitat Politècnica de Catalunya (UPC-BarcelonaTECH), 08034 Barcelona, Spain; 4 Department of Strength of Materials and Structural Engineering, Universitat Politècnica de Catalunya (UPC-BarcelonaTECH), 08034 Barcelona, Spain; miquel.ferrer@upc.edu

**Keywords:** FEA, FEM, impact test, transient analysis, dynamical forces, biomechanical behavior, implant rehabilitation, rehabilitation materials, crown materials

## Abstract

In the literature, many researchers investigated static loading effects on an implant. However, dynamic loading under impact loading has not been investigated formally using numerical methods. This study aims to evaluate, with 3D finite element analysis (3D FEA), the stress transferred (maximum peak and variation in time) from a dynamic impact force applied to a single implant-supported prosthesis made from different materials. A 3D implant-supported prosthesis model was created on a digital model of a mandible section using CAD and reverse engineering. By setting different mechanical properties, six implant-supported prostheses made from different materials were simulated: metal (MET), metal-ceramic (MCER), metal-composite (MCOM), carbon fiber-composite (FCOM), PEEK-composite (PKCOM), and carbon fiber-ceramic (FCCER). Three-dimensional FEA was conducted to simulate the collision of 8.62 g implant-supported prosthesis models with a rigid plate at a speed of 1 m/s after a displacement of 0.01 mm. The stress peak transferred to the crown, titanium abutment, and cortical bone, and the stress variation in time, were assessed.

## 1. Introduction

Currently, implant-supported prostheses are widely used for the rehabilitation of partially and fully edentulous patients. This type of treatment has undergone significant changes in the choice of materials since the first treatments carried out by Brånemark. The use of gold or gold alloys, with and without resin veneering [1,2], has been discarded for economic, esthetic, and functional reasons [3,4,5]. The increase in the price of gold led to the use of much cheaper non-noble metals, although with different mechanical and biological characteristics [3,4,5,6]. The composites and resins used at the end of the last century showed significant deficiencies in esthetics and wear; they were replaced by ceramics and, currently, by zirconia, [6,7,8,9] with different mechanical characteristics. The choice of the material used for implant-supported prosthesis manufacturing is a crucial issue due to the dynamic characteristics of the stomatognathic system.

Static forces are applied from the mandible to the maxilla, without mandibular movements, and the intensity remains constant over time. In contrast, dynamic forces are related to mandibular movements, and the intensity varies with time. The dynamic force magnitude is calculated by multiplying the mass of the moving object and its acceleration in that direction. Static (clenching) and dynamic forces (chewing, swallowing, and eccentric bruxism) occur in the masticatory system [10,11,12,13,14,15]. The literature shows that forces are transferred to a lesser or greater extent to the peri-implant area [16] depending on whether the applied force is static or dynamic [17,18,19,20,21,22,23,24]. Moreover, the results in recent publications showed that static loading, compared with dynamic loading, caused increased stress, which proves the need of transient analysis of dental implants [25,26].

The chosen material for single implant-supported prostheses manufacturing has little relevance in the transmission of static forces, as explained in the Saint-Venant principle, which states that the difference between the effects of two different but statically equivalent loads becomes minimal at sufficiently large distances from the load [27,28,29]. Dynamic forces and the impact of the moving mandible against the maxilla are transferred very differently in single and multiple implant-supported prostheses, depending on the material that the prostheses are made from. Rigid materials, such as zirconia, ceramics, and metals, generate higher dynamic forces [17,19,20,21] than other materials used in veneering prosthetic frameworks (composites, hybrid composites, or resins) or in prosthetic framework manufacturing (carbon fiber, fiberglass, or polyether-ether-ketone (PEEK)), which absorb and dissipate the impact energy with lower dynamic forces [28,29,30,31,32,33,34,35,36,37,38,39,40].

There are different in vitro methods for studying the transmission of static and dynamic forces to the peri-implant area from single and partial implant-supported prostheses made from different materials, such as the use of photoelastic resins [18,29,41,42,43], digital image correlation (DIC) [29,44,45], strain gauges [19,46], loss coefficient (LC) [21], and finite element analysis (FEA) in two (2D FEA) and three dimensions (3D FEA) [22,24,31,47,48,49,50,51]. All of them provide very similar results [29,52,53,54] in terms of stress.

Photoelastic resins allow visualizing the stress generated in the peri-implant area after the application of a static or dynamic force with isochromatic fringes. The color and number of the shown isochromatic fringes indicates the magnitude of the generated stress. Digital image correlation (DIC) is an optical-numerical system using resins with randomly colored microdots, where the displacement of these microdots is calculated after the application of a force, both vertically and horizontally. The magnitude of transferred forces is determined according to the magnitude of the displacements.

Magne et al. [21] used the Periometer (University of Southern California, Los Angeles, CA, USA) to calculate the energy absorbed by prostheses made with different frameworks and veneer materials, such as composite, ceramic, and zirconia. The Periometer is a handheld percussion probe that records the rebound suffered by the object of study, so the energy absorbed by the material can be calculated by subtracting the applied force and the rebound force.

Another system is the use of strain gauges, which are sensors that measure the material strain when loads are applied. Gracis [19] recorded the impact force transmitted by a steel ball rolled along a slope to discs made from different materials. Menini [17,20], used strain gauges to design a device that applied oscillating movements to monolithic prostheses of different materials (gold, zirconia, ceramics, composites, and resins) against an upper dental arch made of a Co-Cr alloy. The force transferred to the crowns (made from different materials) by the simulation of the mandibular movements was recorded.

Dental biomechanics based on finite element analysis (FEA) is attracting huge interest in many areas: biomedical sciences, anthropology and, odontology. However, several shortcomings in FEA modeling exist, mainly due to unrealistic (static) loading imposition [55]. FEA analysis is the most widely used numerical procedure today, since it allows reproducing mechanical behavior under a mechanical load based on the known properties of the material. Density, the Poisson coefficient, and Young’s modulus values can be set in 2D or 3D FEA software, which also includes the depth dimension. Three-dimensional FEA permits the visualization of the stresses on the entire body of the implants. In the consulted dental literature, dynamic FEA studies are still scarce [25,26,55,56,57,58] compared to the large number of existing FEA studies with static loads. Moreover, very few studies that simulate dynamic forces under impact loading using 2D or 3D FEA have been found. Thus, this article is devoted exclusively to the study of the impacts on dental implants, which is minimally covered in the literature.

Knowledge about stress distribution in the peri-implant area may be essential for predicting the survival of dental implants, especially in patients with risk factors such as smoking, poor hygiene habits, previous periodontitis, or predisposing genetic factors [59,60,61,62,63]. These patients present, to a greater or lesser extent, gingival inflammation that may cause peri-implant bone loss [64,65,66,67,68,69,70,71,72]. This peri-implant bone loss may be directly affected by the stress generated in the implant-bone-prosthesis area; the higher the transferred force, the higher the risk of peri-implantitis [21,73,74,75,76,77,78,79]. The amount of cortical bone could also be a factor to be considered when choosing the material for manufacturing the prosthesis, as this cortical bone is poorly vascularized, fragile, rigid, and regenerates slowly [80,81,82,83,84].

Numerous studies have shown, using 2D or 3D FEA, the behavior of implants rehabilitated with single crowns made with different materials. In these studies, all of them used a static force to simulate the oral environment. Our study has aimed to show, using dynamic 3D FEA, the dynamic impact forces related to oral function.

This in vitro study aims to evaluate, with three-dimensional finite element analysis (3D FEA), the stress transferred (time to peak, maximum peak, and variation in time) from an impact, a dynamic force, on a single implant-supported prosthesis made from different restorative materials (metal, metal-ceramic, metal-composite, carbon fiber-composite, PEEK-composite, and carbon fiber-ceramic), applied to the crown, titanium abutment, and cortical mandibular bone.

## 2. Materials and Methods

### 2.1. The Whole Implant Model

The 3D digital model simulated dental rehabilitation on the implants used in this study to evaluate the stress (von Mises stress) on the inner part of the crown, the external part of the neck of the titanium abutment, and the top of the cortical bone, using different implant crowns in a dynamic situation (chewing, swallowing, or eccentric bruxing). This was obtained from the integration of six independently developed models from real elements: (1) the crown, (2) an anti-rotatory abutment, (3) a fixation screw, (4) a single implant-supported prosthesis, (5) a section of the mandibular bone (cortical and cancellous bone), and (6) the plate. Total osseointegration of the implant was considered, assuming a perfect relation between the nodes at the interface of the implants and the bone.

#### 2.1.1. The Crown

In order to obtain a solid model of the crown, a high-resolution 3D Exocad model was imported to SolidWorks. Then, two parts were created within the crown geometry (the core and the esthetic veneering), separated by an inner boundary. The framework and the veneering material were delimited from the single implant-supported prosthesis. The total volume of the crown was 411.5 mm^3^. The framework core accounted for 51.3% of the total crown volume, and the remaining 39.7% was esthetic veneering.

#### 2.1.2. The Abutment and Fixation Screw

The abutment’s function is to join the crown and the implant with a thread mechanism. Also, an anti-rotation system must be available to prevent the relative movement between the implant and the abutment (in this case, a hexagonal anti-rotational system). The abutment and the fixation screw were fully modeled using the CAD software SolidWorks v.2021 (Dassault Systèmes, SolidWorks Corp., Waltham, MA, USA) [85] in order to reduce the typical surfaces of a 3D scanning process to triangular forms, thus maintaining simpler geometries. The abutment used in this study was the MIS implant with an internal hexagonal connection.

#### 2.1.3. The Implant

Accurate measurements of implant geometry were obtained by 3D digital scan (Visual Computing Lab, Pisa, Italy) of a 4.2 × 11.5 mm implant with an internal hexagon (MIS Implants Technology, Bar-Lev, Tel Aviv-Yafo, Israel), which was converted into an STL (Standard Tessellation Language) mesh. Then, it was converted into a solid with the SolidWorks Software (Dassault Systèmes, Vélizy-Villacoublay, France) in order to obtain the measurements of the implant. Finally, it was modeled with the CAD SolidWorks software in order to guarantee more precise geometry and to avoid too many surfaces being shown.

#### 2.1.4. The Mandible

The section of the mandible bone was designed from a sectional image of cone-beam computed tomography (CBCT) (NewTom Giano, Newtom, Imola, Italy). Keypoints were drawn at a fixed distance over the section image of the CT scan in order to transfer it to the computer. The geometry of the mandible could be obtained with SolidWorks software by measuring the distances of the points and calculating the real value through the scanning scale. Two different bounded solids were created over the mandible geometry to apply the mechanical properties of both trabecular and cortical bone.

#### 2.1.5. The Plate

A fixed rigid body with a flat surface was required to simulate impact loads on the tooth during chewing. To this end, a rectangular-shaped plate (*w* = 10, *h* = 12, *e* = 2 mm) was set up to apply the impact load on the three parts of the whole model: the crown, the implant, and the mandible. The initial distance between the plate and the crown was only 0.01 mm. The collision with the plate was frictionless. This means that a zero coefficient of friction was assumed and allowed free sliding. In addition, normal pressure equaled zero if separation occurred.

### 2.2. Material Properties

All materials were modeled as linear elastic isotropic and homogeneous. Young’s modulus and Poisson ratio of each material are shown in Table 1. The mechanical properties of the different materials of the crowns have been provided by the manufacturers.

Abbreviated names of the crown materials are the following: FCOM is a carbon fiber-composite crown, MCER is a metal-ceramic crown, MET is a metal crown alloy (Cr-Co, Mo, and W).

### 2.3. Numerical Methods

**Figure 1:**
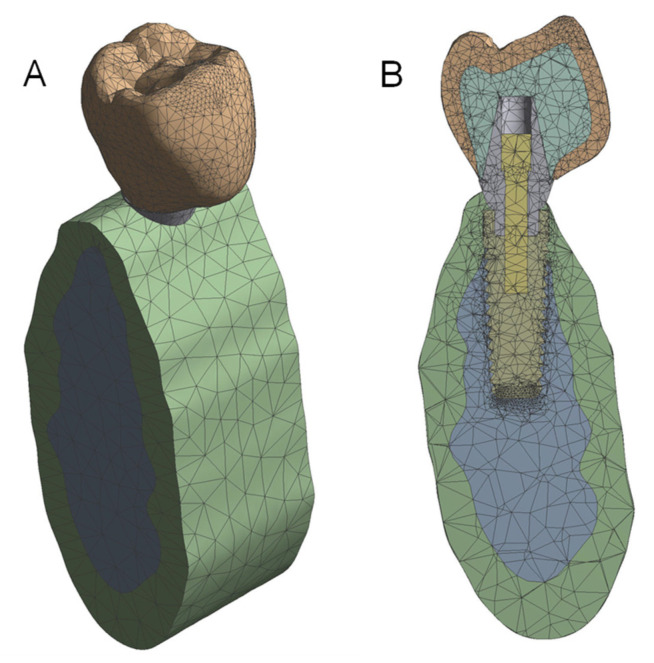
View of the whole 3D FEA model (**A**). Sectional model (**B**).

All independent models were put together by assembly modeling, generating a unique prosthesis-implant-bone model (Figure 1). The geometry was converted to an IGES file, and Ansys Workbench Software (Ansys Inc., Canonsburg, PA, USA) was used to determine the stress transferred to the crown, titanium abutment, and cortical bone before the FEA simulation by the implant-supported prosthesis made from different materials.

The prosthesis-implant-bone model was simulated to collide with a 10 × 12 × 2 mm fixed and rigid plate at a speed of 1 m/s after a displacement of 0.01 mm. For accurate results, the size of the elements is very important. The FEA model had 96,160 nodes and 62,606 elements to simulate the real models (prosthesis, implant, and bone) (see Figure 1). Young’s modulus, Poisson’s coefficient, and density were assigned to each material used in the manufacturing of the implant-supported prosthesis: CoCr (MET), CoCr-Ceramic (MCER), CoCr-Composite (MCOM), Carbon Fiber-Composite (FCOM), PEEK-Composite (PKCOM), Carbon Fiber-Ceramic (FCCER), the titanium abutment, and the cortical bone of the model (Table 1). For the FEA, all materials were considered isotropic and homogeneous, displacements were only in the vertical direction, perfect osseointegration was assumed, the impact was carried out on a rigid object (plate), and, finally, the collision was frictionless.

#### 2.3.1. Mesh Definition

Before performing the simulation with the finite element method, the mesh size and the element type must be defined. The accuracy of the results depends directly on the size of the elements. The smaller the elements, the more accurate the solution. Therefore, small elements were used in order to improve precision. However, this affected the computational time. While CPU time is not that important in static analyses, it is crucial in transient dynamic analyses.

The solid 3D element SOLID187 (Ansys Inc., Canonsburg, PA, USA) [85] was used, with 10 nodes and quadratic interpolation functions that are more suitable for irregular geometries. The element had three degrees of freedom per node, i.e., the three translations in the global coordinate directions *x*, *y*, *z*. Surface-to-surface contact was defined with the element CONTA174.

In the process of creating the mesh, a refinement process was carried out in order to obtain a stable solution independent of the mesh size, especially around the impact zone, thereby ensuring high accuracy in this area. Therefore, as this refinement had been done, it was not necessary to use an area to obtain an average solution, since the nodal solution was especially accurate. Thus, the corresponding mesh was then considered to be optimal.

In addition, Ansys software performs control of the aspect ratio systematically. The accuracy of the results depends directly on the size of the finite element mesh. The smaller the mesh, the more accurate the solution obtained. Near the loading point and the threaded part, where higher accuracy was needed, the size was 0.2 mm, but in the other parts it was larger, from 0.5 to 2 mm. Even if the different parts of the implant are assembled together, the finite element results can be analyzed independently. Six solids were considered individually: the crown, the abutment, the implant, the fixation screw, the mandible, and the plate.

#### 2.3.2. Simulation Time

Regarding simulation time, 0.4 ms were simulated. The number of substeps is the number of intervals into which the simulation time is divided. That is to say, the calculation time-step between one instant to the next. If they are too small, the computing time increases considerably and, if they are set too high, the accuracy of the time-history response decreases. A value of 53 substeps, i.e., a time-step of 7.55 μs, was found to be reasonable.

## 3. Results

### 3.1. Stress Results

**Figure 2:**
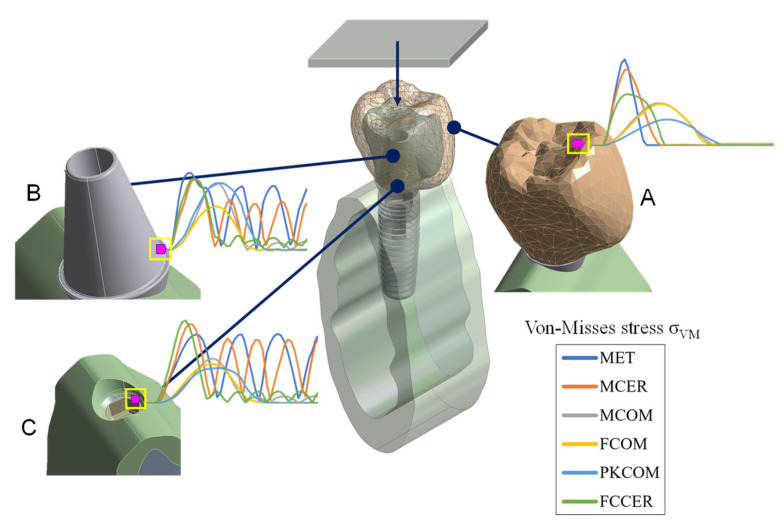
The nodes selected for numerical simulation. (**A**) Sectional view of the 3D FEA model at the crown node. (**B**) The abutment node. (**C**) The node on top of the cortical bone.

The von Mises stress value (obtained from a Cauchy stress tensor) was calculated over time in the dynamic FEA simulation and compared for each node (Figure 2) in a time interval of 0.4 ms. The stress peak values in the crown, titanium abutment, and cortical bone are summarized in Table 2.

**Figure 3:**
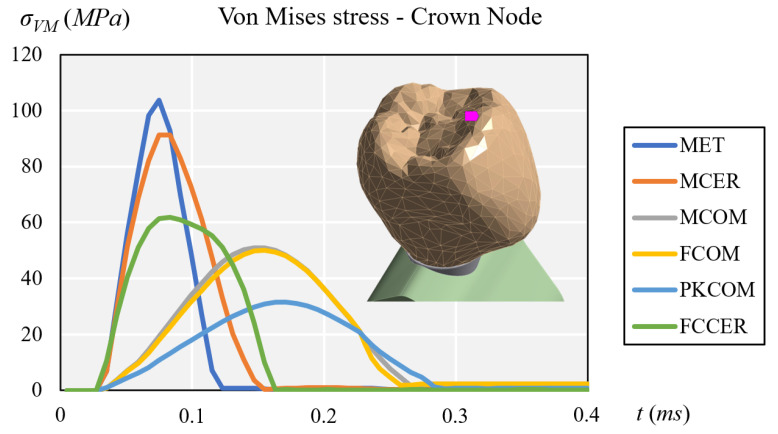
Comparison of equivalent von Mises stress at the crown node.

At the crown node (Figure 3) the maximum peaks were found at the MET and MCER crown, followed by that at FCCER. The lower values were found at MCOM and FCOM, and the lowest at the PKCOM crown.

**Figure 4:**
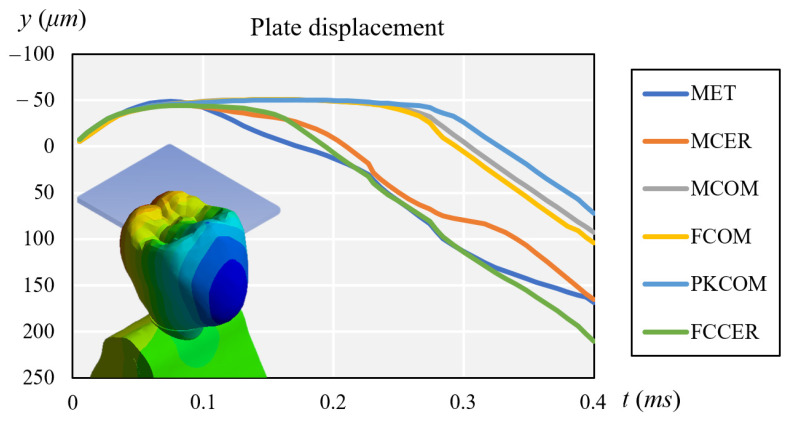
Comparison of plate displacement for each crown after impact.

At the same time, Figure 4 compares the displacement of each crown during impact.

**Figure 5:**
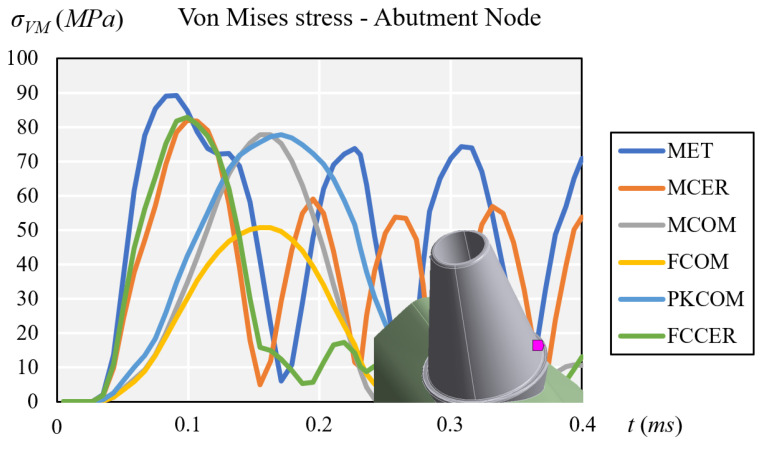
Comparison of equivalent von Mises stress at the titanium abutment node.

All the crowns except FCOM showed high peak intensity values at the titanium abutment node (Figure 5). MET and MCER showed higher stress rebound over time, while MCOM, FCOM, PKCOM, and FCCER showed no rebound peaks after the impact (See Figure 5).

**Figure 6:**
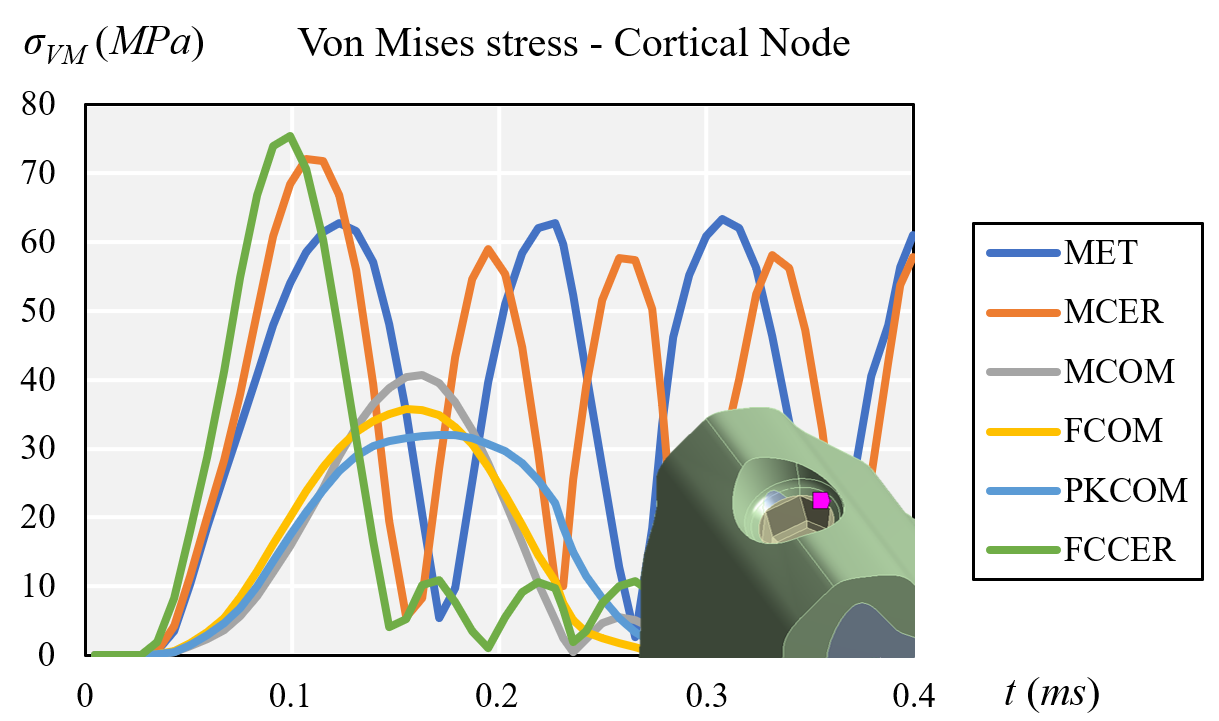
Comparison of equivalent von Mises stress at the cortical node.

Composite-veneered implant-supported prostheses (MCOM, FCOM, and PKCOM) generated lower stress peaks at the cortical bone than ceramic-veneered (MCER and FCCER) or all-metal (MET) implant-supported prostheses. The implant-supported ceramic-veneered (MCER and FCCER) or all-metallic (MET) prostheses exhibited a more significant and earlier stress peak on the cortical bone than those veneered with composite (MCOM, PKCOM, and FCOM) (Figure 6). The highest stress rebound peaks happened in MET and MCER implant-supported prostheses. Implant-supported prostheses made of carbon fiber-ceramic (FCCER) showed the highest maximum peak of stress, but it dissipated quickly with rebound peaks of lower intensity. A rapid reduction in stress was observed in implant-supported prostheses veneered with composite (MCOM, PKCOM, and FCOM) and in those made with carbon fiber-ceramic (FCCER) (Figure 6).

### 3.2. Elastic Failure Test

A failure test was carried out to see if the dental implants could withstand the mechanical conditions to which they were subjected. Elastic failure criteria establish different approaches for different materials. In this case, the von Mises or maximum elastic distortion energy criterion was used. This criterion says that a structural element fails when at some point the distortion energy per unit volume exceeds a certain threshold. In stress terms, this means that the equivalent stress at a point, which is the von Mises stress, cannot exceed the elastic limit or the yield strength of the material, *σ_y_*:

(1) $$\sigma_{VM}\leq \sigma_{y}$$

Consequently, research on the elastic limits of the different materials was needed. After obtaining the values, a comparison was made for each model of the dental implant with each of the studied nodes used before. In Table 3, the yield stress, *σ_y_*, and the maximum value of stress, *σ_VM_*_max_, are compared for each material and node. In this table, we can observe how the largest stresses occurred in the most rigid models.

In order to prevent uncertainties that may occur when real loads act on the implant, a safety factor is used. The safety factor is defined as the ratio between the yield strength of the material and the maximum value of von Mises equivalent stress. A usually applied Safety Factor is 1.5.

(2) $$\gamma_{SF}=\frac{\sigma_{y}}{\sigma_{VM}}=1.5.\;$$

Taking yield strength as the 100% value and rearranging Equation (2):

(3) $$\sigma_{VM}\leq \frac{100\%\cdot \sigma_{y}}{1.5}=66.67\%\cdot \sigma_{y}$$

**Figure 7:**
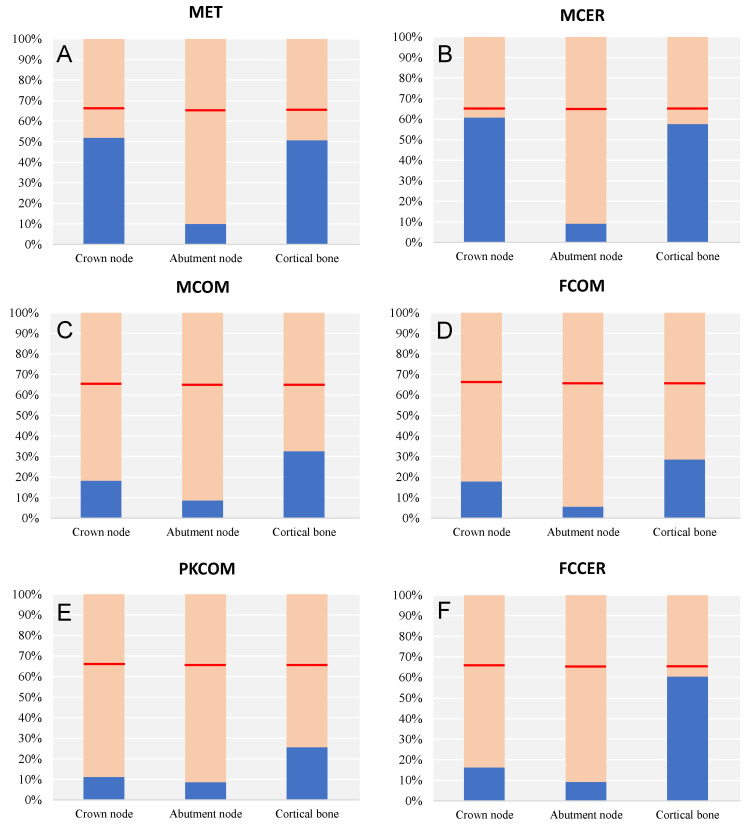
Comparison of the yield strength ratio depending on the node and crown material.

Figure 7 shows the yield strength ratio for each material and node. The red line indicates the 66.67% value of yield stress. 

There was no elastic failure in any model, since all von Mises stresses were below the elastic limit, taking an arbitrary safety factor of 1.5. In the bar plots, we can observe how the von Mises stresses did not surpass 66.67% of the yield stress (red line). The most rigid models with the highest von Mises stresses were the ones closest to the 66.67% of the yield stress of each material.

In summary, the assumption of linearity of the behavior of materials was fulfilled in the studied model, and the calculated stresses were below the yielding limits of the materials, so we can consider that there was no plasticization.

**Figure 8:**
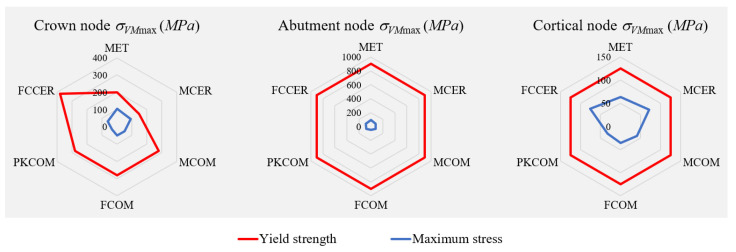
Spider plots comparing the yield strength of the materials and the maximum von Mises stress obtained for each node.

The spider plots of Figure 8 show a comparison between the yield strength, *σ_y_*, of the materials and the maximum values of von Mises stress, *σ_VM_*_max_, obtained in the numerical simulations for each node.

## 4. Conclusions

Denture forces, such as those from chewing, are transferred to implants and cause stress in the bone and the implant. That is why it is important to study the stresses (or strains) transferred to the implant and the bone in situations of maximum stress, modeled by dynamic forces under impact loading.

It can be concluded from the results of this study that the stress transferred to the crown, the abutment, and the peri-implant bone by an impact load on an implant-supported prosthesis varies according to the rigidity of the material and whether it is used as a framework or veneering material. It can also be stated that the more elastic material used for the crown, the lower the stresses generated in the bone. Too much stress induces bone resorption, which ultimately causes loosening of the implant, and overstrain can instigate bone failure. It turns out that the use of PEEK or carbon fibers as framework materials made stress dissipate faster than when using metal at the bone. By using these materials that can absorb and/or dissipate the stress transferred to the implant, we can reduce the risk of having bone resorption around the implant.

Therefore, with the use of more elastic materials that can better dissipate the impact energy and reduce the stress transferred to the implant, the risk of having bone resorption around the implant can also be reduced, especially in patients at risk of gingival inflammation that may cause peri-implant bone loss.

## Figures and Tables

**Table 1:** Properties of materials used in the prothesis and the bone (trabecular and cortical).

Kerrypnx	Material Name	Manufacturer	*E* Young Modulus (MPa)	*v* Poisson Ratio	*ρ* Density (g/cm^3^)
**Crown**	**FCOM**				
	Carbon fiber-composite	[86]			
	BioCarbon Bridge fibers	Micro Medica	300,000	0.3	1.40
	Composite BioXfill	Micro Medica	22,000	0.3	8.30
	**MCER**				
	Metal-ceramic	[87,88]			
	Co-Cr alloy	Renishaw	208,000	0.31	8.90
	Ceramic VMK 95	Vita	69,000	0.28	2.50
	**MCOM**				
	Metal-composite	[86,87]			
	Co-Cr alloy	Renishaw	208,000	0.31	8.90
	Composite BioXfill	Micro-Medica	22,000	0.3	8.30
	**MET**	[89]			
	Full metal				
	Co-Cr Alloy, Mo, W	Heraeus Kulzer	208,000	0.31	8.90
	**FCCER** Carbon fiber-ceramic	[86,90]			
	Carbon Fiber Bridge	Micro-Medica	66,000	0.3	1.4
	Ceramic IPS e.max	Ivoclar Vivadent	95,000	0.2	2.5
	**PKCOM** PEEK-composite	[86,91]			
	PEEK Optima	Invibio	4100	0.36	1.3
	Composite BioXfill	Micro-Medica	22,000	0.3	8.30
**Implant**	Ti-6-Al-4V ELI	MIS [92]	113,800	0.34	4.43
**Bone**	Cortical bone	[93,94]	15,000	0.3	1.79
	Trabecular bone	[93]	500	0.3	0.45

**Table 2:** Maximum equivalent von Mises stress transferred to the crown, the titanium abutment, and the cortical bone by the different prosthesis materials.

**Node/Material**	**Maximum von Mises Stress *σ_VM_*_max_ (MPa)**					
	**MET**	**MCER**	**MCOM**	**FCOM**	**PKCOM**	**FCCER**
Crown	103.81	91.18	51.05	49.98	31.51	61.82
Abutment	89.27	81.91	77.82	50.80	77.78	82.80
Cortical	63.35	72.06	40.71	35.70	32.05	75.46

**Table 3:** Comparison of the yield stress, *σ_y_*, and the maximum value of stress, *σ_VM_*_max_, for each material and node.

Material	Node	Yield Strength *σ_y_* (MPa)	Maximum von Mises *σ_VM_*_max_ (MPa)
MET	Crown	145–270	103.81
	Abutment	880–920	89.27
	Cortical	100–150	63.35
MCER	Crown	150	91.18
	Abutment	880–920	81.91
	Cortical	100–150	72.06
MCOM	Crown	280	51.05
	Abutment	880–920	77.82
	Cortical	100–150	40.71
FCOM	Crown	280	49.99
	Abutment	880–920	50.80
	Cortical	100–150	35.70
PKCOM	Crown	280	31.51
	Abutment	880–920	77.78
	Cortical	100–150	32.05
FCER	Crown	380	61.82
	Abutment	880–920	82.80
	Cortical	100–150	75.46

## Data Availability

Not applicable.

## Abbreviations

MET	CoCr Metal
MCOM	CoCr Metal-Composite
MCER	CoCr Metal-Ceramic
FCOM	Carbon Fiber-Composite
PKCOM	PEEK-Composite
FCCER	Carbon Fiber-Ceramic
